# P05.41. Development and evaluation of group medical visits for medically underserved women with chronic pelvic pain

**DOI:** 10.1186/1472-6882-12-S1-P401

**Published:** 2012-06-12

**Authors:** M Chao, P Abercrombie

**Affiliations:** 1 Univ of California, San Francisco, Osher Center for Integrative Med, San Francisco, USA

## Purpose

Chronic pelvic pain (CPP) severely impacts quality of life and is one of the most common medical problems among women. Effective management of CPP is needed, particularly for underserved women with limited access to quality care. We developed and evaluated group medical visits for women with CPP drawing from: (1) the Centering model, an innovative approach to group-based healthcare across the lifecourse; and (2) integrative medicine emphasizing quality of life through patient-centered care.

## Methods

Curriculum for ten monthly group visits of CenteringIMPPACT (Centering and Integrative Medicine for chronic Pelvic PAin Comprehensive Treatment) included mind-body approaches to managing CPP. A pilot study was conducted among patients with CPP at a safety net hospital. Qualitative and quantitative data were collected at baseline and three follow-up time points to assess feasibility, acceptability, and preliminary data on efficacy.

## Results

CenteringIMPPACT participants (n=12) were on average 42 years of age; 58% were African American; 42% had attended some college; and 83% had household incomes of less than $35,000; 42% reported being in fair or poor health. From baseline to 3-month follow-up, average number of days that women reported limitations of usual activities decreased from 24 to 15. Health-related quality of life improved slightly; average life interference due to CPP decreased from 2.64 to 2.43. Mental health did not improve: scores on the Patient Health Questionnaire PHQ-9 for Depression increased from 11.25 to 12.43. Based on qualitative data, participants experienced healthcare empowerment through learning to advocate for themselves and increased support and hope from the groups.

## Conclusion

Preliminary data from this study indicates some improvements in functional activities and health-related quality of life among medically underserved women with CPP. Group medical visits are a viable model of comprehensive care for CPP in safety net healthcare settings. Additional data are needed to further evaluate the effects of CenteringIMPPACT.

